# Enhanced detection of *Pythium insidiosum* via lipid profiling with matrix-assisted laser desorption ionization time of flight mass spectrometry

**DOI:** 10.1186/s43008-024-00163-8

**Published:** 2024-11-01

**Authors:** Nichapat Yurayart, Paisan Jittorntam, Yothin Kumsang, Thidarat Rujirawat, Atisak Jiaranaikulwanich, Theerapong Krajaejun

**Affiliations:** 1 Department of Pathology, Faculty of Medicine, Ramathibodi Hospital, Mahidol University, 270 Rama 6 Road, Bangkok, 10400 Thailand; 2 Research Center, Faculty of Medicine, Ramathibodi Hospital, Mahidol University, Bangkok, Thailand

**Keywords:** *Pythium insidiosum*, Pythiosis, Lipid profiles, MALDI-TOF, Diagnosis

## Abstract

Pythiosis is a severe disease in humans and animals globally, caused by the pathogenic oomycete *Pythium insidiosum*. Early and accurate detection is crucial for effective treatment, but traditional diagnostic methods have limitations. This study presents an alternative approach using Matrix-Assisted Laser Desorption Ionization Time of Flight Mass Spectrometry (MALDI-TOF MS) for lipid profiling to efficiently identify *P. insidiosum*. The study involved extracting microbial lipid components using optimized chloroform: methanol biphasic method and creating a lipid profile database with samples from 30 *P. insidiosum* isolates and 50 various fungi. The methodology was validated on 25 blinded samples for assay detection performance. Unique lipid profiles allowed species-specific identification with high efficiency: scores ≥ 2.682 indicated *P. insidiosum*, scores ≤ 2.512 suggested fungi, and scores in between pointed to other oomycetes. The assay demonstrated sensitivity, specificity, and accuracy of 100%, 80%, and 88%, respectively, for detecting *P. insidiosum*. The limited detection specificity was due to false positive samples from closely related *Pythium* species, which are not a significant clinical concern. The findings show that MALDI-TOF MS lipid profiling can efficiently identify *P. insidiosum*, offering significant advantages in sample preparation, stability, and reproducibility over protein profile-based methods. This study marks the first instance of lipid profiles being reported for *P. insidiosum*, paving the way for clinical use in improving accurate detection and facilitating timely treatment interventions.

## Introduction

*Pythium insidiosum*, a critical fungal-like microorganism in the class of Oomycetes, thrives in stagnant water environments such as ponds and rice fields (Mar Htun et al. 2021). Characterized by sparsely septate hyphae, its morphology closely resembles that of filamentous fungi (De Cock et al. 1987). Recent studies have revealed the existence of 4 genotypes of *P. insidiosum,* designated as clades I through IV, with a global distribution, particularly in tropical and subtropical regions, including the United States, South America, Australia, India, and Thailand (Gaastra et al. 2010; Yolanda and Krajaejun 2022). This organism is notorious for causing pythiosis, a severe disease affecting humans, horses, and dogs, with sporadic cases in cats, cattle, and spectacled bears (Schurko et al. 2003; Krajaejun et al. 2006; Nguyen et al. 2022). Transmission occurs when a motile zoospore directly encounters humans or animals, significantly risking those with underlying hematological conditions such as thalassemia, particularly in cases of vascular pythiosis (Mendoza et al. 1993; Krajaejun et al. 2006; Laohapensang et al. 2009; Keoprasom et al. 2013). The manifestation of the disease varies, with vascular and ocular forms being prevalent in humans, whereas animals frequently suffer from cutaneous/subcutaneous and gastrointestinal infections (Thianprasit et al. 1996; Yolanda and Krajaejun 2022). Early and accurate diagnosis is paramount to ensure effective treatment and improve patient outcomes.

Historically, identifying *P. insidiosum* has relied on morphological analysis, serological assays, and molecular detection techniques. However, these methods come with limitations, including the difficulty of morphologically distinguishing *P. insidiosum* from clinically relevant fungal pathogens, the potential for immunological cross-reactivity with some pathogenic fungi, and the carryover of genetic materials during the assay analysis, which can lead to false-positive results (Rotchanapreeda et al. 2021). These challenges necessitate expert interpretation and can be time-consuming. In recent years, matrix-assisted laser desorption ionization-time of flight mass spectrometry (MALDI-TOF MS) has emerged as a promising tool for microbial identification, biotyping, and evaluating drug resistance by analyzing cellular components such as proteins (Stevenson et al. 2010). This technology has been applied to identify *P. insidiosum* (Krajaejun et al. 2018; Mani et al. 2019). Notably, the identification capabilities of MALDI-TOF MS extend beyond proteins to include lipids, which have recently been identified as species-specific markers that can offer a robust basis for microbial identification (Solntceva et al. 2020).

This study marks the first instance of lipid profiles being reported for *P. insidiosum*, paving the way for their use as an efficient identification method. The exploration and validation of lipid profiles via MALDI-TOF MS enhance our understanding of *P. insidiosum* and underscore the potential of lipid-based biomarkers for microbial identification practices. By focusing on the lipidomic approach, we can achieve an accurate and rapid identification of *P. insidiosum*, thereby facilitating timely and appropriate treatment interventions.

## Materials and methods

### Microorganisms and culture condition

Lipid profiles were generated from *Pythium insidiosum* (comprising 8 clade-I, 11 clade-II, and 11 clade-III isolates) and various fungi (n = 50) (Tables 1 and 2). This study also included 25 blinded samples from 10 *P. insidiosum* isolates and 15 other filamentous organisms (Table 3) to validate lipid profile-based microbial identification. The identity of each organism was assigned based on the morphologies and rDNA ITS sequence analysis (Krajaejun et al. 2018). In preparing hyphal material for lipid extraction, all organisms were grown on 1% Sabouraud dextrose agar (SDA) at 25 °C for 7 days. Each organism (100 mg) was harvested and stored overnight in 300 µl of methanol at − 30 °C. After removing methanol, the hyphal mat was collected and kept at − 30 °C until use.

**Table 1:** *Pythium insidiosum* database strains for generating the lipid profiles by MALDI-TOF MS analysis and setting a cutoff identification score

*P. insidiosum*		Source	Country of origin	Accession (rDNA ITS)	MALDI-TOF MS mapping against *P. insidiosum* database strains				
Genotype	Strain				Self-mapped score	Best non-self mapped strain	Identification score	Worst non-self mapped strain	Identification score
Clade-I (n = 8)	Pi001C1	Equine	Costa Rica	LC199875	2.825	Pi073C1	2.758	Pi045C3	2.516
	Pi008C1	Equine	Costa Rica	AB898107	2.836	Pi047C3	2.772	Pi073C1	2.082
	Pi009C1	Equine	Brazil	AB971181	2.802	Pi052C2	2.725	Pi008C1	2.243
	Pi010C1	Human	USA	AB898108	2.839	Pi008C1	2.754	Pi085C2	2.518
	Pi063C1	Equine	Brazil	LC550294	2.822	Pi052C2	2.717	Pi074C1	2.269
	Pi072C1	Equine	Brazil	LC801545	2.740	Pi001C1	2.732	Pi009C1	2.277
	Pi073C1	Canine	Brazil	LC801546	2.799	Pi056C2	2.719	Pi072C1	2.353
	Pi074C1	Canine	Thailand	LC801547	2.839	Pi001C1	2.771	Pi044C3	2.549
Clade-II (n = 11)	Pi023C2	Human	Thailand	AB898115	2.81	Pi001C1	2.765	Pi081C3	2.434
	Pi026C2	Human	Thailand	AB898117	2.837	Pi073C1	2.724	Pi084C3	2.445
	Pi036C2	Equine	Australia	LC199883	2.832	Pi047C3	2.779	Pi039C2	2.198
	Pi039C2	Equine	Japan	LC199885	2.831	Pi088C3	2.692	Pi072C1	2.352
	Pi040C2	Mosquito larva	India	LC199886	2.844	Pi087C2	2.682	Pi074C1	2.297
	Pi052C2	Human	Thailand	LC199888	2.817	Pi085C2	2.728	Pi088C3	2.338
	Pi056C2	Equine	Thailand	LC801544	2.840	Pi001C1	2.789	Pi077C3	2.161
	Pi082C2	Rice field	Thailand	LC556033	2.827	Pi056C2	2.784	Pi045C3	2.079
	Pi085C2	Rice field	Thailand	LC556036	2.807	Pi083C3	2.773	Pi088C3	2.392
	Pi087C2	Rice field	Thailand	LC556038	2.807	Pi082C2	2.734	Pi056C2	2.010
	Pi090C2	Rice field	Thailand	LC556041	2.811	Pi008C1	2.732	Pi063C1	2.085
Clade-III (n = 11)	Pi044C3	Human	Thailand	AB971185	2.843	Pi056C2	2.744	Pi023C2	2.299
	Pi045C3	Human	Thailand	AB971186	2.841	Pi056C2	2.725	Pi050C3	2.374
	Pi047C3	Human	Thailand	AB971188	2.833	Pi049C3	2.753	Pi001C1	2.336
	Pi049C3	Human	Thailand	AB898127	2.842	Pi036C2	2.792	Pi045C3	2.397
	Pi050C3	Human	U.S.A	AB971190	2.832	Pi047C3	2.796	Pi073C1	2.141
	Pi077C3	Zoo pond	Thailand	LC556017	2.832	Pi083C3	2.793	Pi039C2	2.365
	Pi081C3	Rice field	Thailand	LC556032	2.800	Pi077C3	2.684	Pi010C1	2.210
	Pi083C3	Rice field	Thailand	LC556034	2.843	Pi088C3	2.782	Pi010C1	2.403
	Pi084C3	Rice field	Thailand	LC556035	2.804	Pi056C2	2.751	Pi074C1	2.147
	Pi088C3	Rice field	Thailand	LC556039	2.815	Pi083C3	2.803	Pi085C2	2.440
	Pi089C3	Rice field	Thailand	LC556040	2.756	Pi082C2	2.727	Pi077C3	2.040

**Table 2:** Control fungal organisms for generating the lipid profiles by MALDI-TOF MS analysis and setting a cutoff identification score

Microorganisms		Best mapped *P. insidiosum* strain	Identification score
Hyaline fungi (n = 31)	*Aspergillus flavus* RA079	Pi081C3	2.102
	*Aspergillus flavus* RA082	Pi047C3	2.337
	*Aspergillus flavus* RA087	Pi047C3	2.345
	*Aspergillus flavus* RA112	Pi081C3	2.215
	*Aspergillus flavus* RA115	Pi047C3	2.243
	*Aspergillus fumigatus* RA018	Pi045C3	2.430
	*Aspergillus fumigatus* RA019	Pi074C1	2.212
	*Aspergillus fumigatus* RA020	Pi084C3	2.400
	*Aspergillus fumigatus* RA032	Pi036C2	2.424
	*Aspergillus fumigatus* RA034	Pi077C3	2.378
	*Aspergillus nidulans* RA009	Pi050C3	2.293
	*Aspergillus niger* RA002	Pi056C2	2.403
	*Aspergillus niger* RA004	Pi056C2	2.394
	*Aspergillus terreus* RA025	Pi056C2	2.502
	*Aspergillus terreus* RA050	Pi073C1	2.426
	*Fusarium* sp. F2303	Pi063C1	1.792
	*Fusarium* sp. F28071	Pi073C1	2.440
	*Fusarium* sp. F8877	Pi050C3	2.218
	*Fusarium* sp. RA014	Pi063C1	1.745
	*Fusarium* sp. RA015	Pi073C1	2.285
	*Fusarium* sp. RA059	Pi073C1	1.880
	*Fusarium* sp. RA063	Pi073C1	2.394
	*Fusarium* sp. RA086	Pi073C1	2.245
	*Fusarium* sp. RA127	Pi073C1	2.330
	*Fusarium* sp. WF6741	Pi073C1	1.917
	*Paecilomyces variotii* RA140	Pi036C2	2.310
	*Penicillium* sp. F9856	Pi044C3	1.957
	*Penicillium* sp. F9857	Pi045C3	1.897
	*Penicillium* sp. F9871	Pi044C3	2.126
	*Penicillium* sp. F9913	Pi077C3	1.963
	*Sarocladium* sp. RA076	Pi083C3	2.512
Dematiaceous fungi (n = 5)	*Scedosporium apiospermum* RA033	Pi050C3	2.281
	*Scedosporium apiospermum* RA055	Pi077C3	2.291
	*Scedosporium apiospermum* RA108	Pi085C2	1.771
	*Scedosporium apiospermum* RA150	Pi036C2	2.364
	*Scedosporium apiospermum* RA151	Pi036C2	2.116
Mucorales fungi (n = 6)	*Mucor* sp. F9534	Pi085C2	2.381
	*Mucor* sp. RA172	Pi077C3	2.368
	*Rhizopus* sp. F9059	Pi073C1	2.480
	*Rhizopus* sp. RA049	Pi073C1	2.433
	*Rhizopus* sp. RA065	Pi073C1	2.179
	*Saksenaea* sp. P16-6918	Pi077C3	2.233
Dermatophyte fungi (n = 8)	*Microsporum canis* RA017	Pi050C3	2.112
	*Microsporum canis* RA053	Pi085C2	2.078
	*Microsporum canis* RA103	Pi050C3	2.305
	*Microsporum canis* RA129	Pi050C3	2.327
	*Nannizzia gypsea* RA038	Pi083C3	2.017
	*Trichophyton mentagrophytes* RA104	Pi044C3	2.234
	*Trichophyton mentagrophytes* RA168	Pi010C1	2.321
	*Trichophyton rubrum* RA170	Pi044C3	2.074

**Table 3:** Validation of lipid-based MALDI-TOF MS analysis for identification of *P. insidiosum* using blinded lipid extract samples

Sample ID	Blinded organisms	Identification score	Identification score-based interpretation^a^
UN-01	*P. insidiosum*	2.728	*P. insidiosum*
UN-02	*P. insidiosum*	2.722	*P. insidiosum*
UN-03	*P. insidiosum*	2.759	*P. insidiosum*
UN-04	*P. insidiosum*	2.719	*P. insidiosum*
UN-05	*P. insidiosum*	2.737	*P. insidiosum*
UN-06	*P. karlingii*	2.610	Other oomycetes
UN-07	*P. insidiosum*	2.705	*P. insidiosum*
UN-08	*P. rhizo-oryzae*	2.663	Other oomycetes
UN-09	*P. insidiosum*	2.738	*P. insidiosum*
UN-10	*P. insidiosum*	2.778	*P. insidiosum*
UN-11	*P. insidiosum*	2.768	*P. insidiosum*
UN-12	*P. insidiosum*	2.737	*P. insidiosum*
UN-13	*Mucor* sp.	2.488	Fungus
UN-14	*T. rubrum*	1.687	Fungus
UN-15	*Rhizopus* sp.	2.453	Fungus
UN-16	*Penicillium* sp.	2.124	Fungus
UN-17	*P. catenulatum*	2.718	*P. insidiosum*^b^
UN-18	*Fusarium* sp.	2.446	Fungus
UN-19	*P. catenulatum*	2.720	*P. insidiosum*^b^
UN-20	*P. aphanidermatum*	2.689	*P. insidiosum*^b^
UN-21	*N. gypsea*	2.277	Fungus
UN-22	*S. apiospermum*	2.364	Fungus
UN-23	*M. canis*	2.275	Fungus
UN-24	*P. variotii*	2.223	Fungus
UN-25	*Saksenaea* sp.	2.276	Fungus

^a^Cutoff identification score for *P. insidiosum* is ≥ 2.682, and for fungus is ≤ 2.512

^b^Misidentification of another organism to *P. insidiosum*

### Extraction of microbial lipid components

Lipid components were extracted from each organism using the previously described chloroform: methanol biphasic method with some modifications (Folch et al. 1957; Wong et al. 2019). In brief, hyphae were washed once with 1 mL of liquid chromatography-mass spectrometry (LC–MS) grade water (Merck, USA) before transferring to a 2-ml screw-cap tube containing 100 mg of glass beads (710–1180 mm in diameter; Sigma, USA) and 300 µl of LC–MS water. The hyphae were homogenized using a Tissue Lyzer Retsch MM301 (Qiagen, Germany) at 30 Hz for 2 min, transferred to a 1.5-mL tube, and centrifuged (3,000 × g) at 4 °C for 2 min. The supernatant was discarded, and 200 µl of chloroform and methanol mixture [2:1 (v/v) ratio] was added to the sample before vortexing at high speed (3,200 rpm) for 2 min, incubating at room temperature for 30 min, and centrifuging (3,000 × g) at 4 °C for 3 min. When the suspension was separated into the top (methanol) and bottom (chloroform) layers with hyphal debris in between, lipid components (dissolved in chloroform) were gently collected and transferred to a new 1.5-mL tube for a same-day MALDI-TOF MS analysis.

### Generation of lipid profiles by MALDI-TOF MS

The extracted lipid component-containing chloroform (0.5 µl) was spotted onto a MALDI-TOF AnchorChip target plate (Bruker Daltonics, Germany) in 40 replicates for generating an in-house lipid profile database. The angiotensin II (Bruker Daltonics, Germany), which has a similar mass/charge (m/z) ratio as lipids, was used to calibrate the mass spectrometer following the manufacturer’s instructions. After the spotted sample was dried, 0.5 µl of matrix solution (5 mg/mL 2, 5-dihydroxybenzoic acid (DHB) in 70% methanol and 0.1% trifluoroacetic acid) was pipetted onto each sample. When the matrix solution was dried, each sample was analyzed using an UltrafleXtreme mass spectrometer (Bruker Daltonics, Germany), using the following settings: positive linear; laser frequency, 60 Hz; ion source-1 voltage, 25 kV; ion source-2 voltage, 24 kV; and lens voltage, 7.0 kV. An individual spot was measured using 50 sets of 200 laser shots in different areas. The signal generated from each lipid sample was subtracted from that of the dried matrix without a spotted sample to eliminate the background mass peaks. The obtained spectra were recorded at m/z ratios ranging from 400 to 1,200.

### Main spectral profiles and microbial identification score

Mass spectra used to construct the lipid profile database were selected based on the established criteria of the National Institutes of Health (NIH) mold and yeast proteomic database. These criteria include each spectrum containing peaks with a resolution of more than 500 using the FlexAnalysis software version 3.0 (Bruker Daltonics, Germany) (Stevenson et al. 2010; Lau et al. 2013). From the 40 mass spectra generated per organism**,** 24 that best represented the organism's characteristics were chosen for constructing a main spectral profile (MSP) and calculating an identification of log (score) value (which is a logarithmic score referred to in short as “identification score”) for microbial identification using the Biotyper OC software version 3.1 (Bruker Daltonics, Germany).

In the performance assessment of the identification score, 25 blinded lipid samples extracted from *P. insidiosum* and other filamentous microorganisms (Table 3) were analyzed in 5 replicates using the MALDI-TOF MS, as mentioned above. The highest score that was obtained was used to interpret the result. To evaluate the performance of the identification score and selected cutoff values, detection sensitivity (the proportion of true positive results), specificity (the proportion of true negative results), and accuracy (the proportion of correct results) were calculated. These calculations were performed using the MedCalc software (MedCalc 2024).

### Impact of growth conditions and storage durations on extracted lipids

Three *P. insidiosum* isolates (strains Pi009C1, Pi085C2, and Pi083C3) representing all genotypes were grown at 25 °C under 4 different culture conditions: (i) 1% SDA for 3 days, (ii) 1% SDA for 5 days, (iii) 1% SDA for 7 days, and (iv) Brain–heart infusion agar (BHI) for 7 days. Lipid components were extracted from each organism and analyzed using MALDI-TOF MS in 5 replicates. The resulting mass spectral data were compared against the in-house *P. insidiosum* lipid profile database. For the stability testing, lipid samples freshly prepared from 3 organisms (unknown sample numbers UN-08, UN-10, and UN-11) were stored at − 30 °C for 14, 21, and 42 days before proceeding with MALDI-TOF MS analysis. Mass spectra were generated for each sample in 5 replications and compared against the *P. insidiosum* lipid profile database.

### Phylogenetic analysis and biotyping of *P. insidiosum* lipids

The rDNA ITS sequences of the *P. insidiosum* isolates used to generate the in-house lipid profile database (Table 1) were recruited for constructing a phylogenetic tree. *P. catenulatum* and *P. rhizo-oryzae* served as the outgroup. All sequences were aligned and curated using the built-in MUSCLE and BMGE algorithms in the NGPhylogeny.fr web-based tool (Lemoine et al. 2019). The phylogenetic tree was generated using MEGA v.11.0 with the Neighbor-Joining algorithm and 1,000 bootstrap replications.

The MSPs of the same set of *P. insidiosum* isolates were adopted for generating a hierarchical clustering tree using MATLAB version 7.1 (Math-Works), MALDI Biotyper (Bruker Daltonics, Germany), and the software default settings. A principal component analysis (PCA) was performed using the lipid mass spectra to differentiate the microorganisms of various species and genotypes. The accuracy of PCA was checked by 3 statistical models [i.e., genetic algorithms (GA), supervised neural network (SNN), and quick classifier (QC)], which were demonstrated as the percentages of recognition capacity (RC) and cross-validation (CV) values. The highest value determined the most suitable model.

## Results and discussion

### Selection of a lipid extraction method

Lipid components can be extracted using various methods. Among them, the Folch method is widely applicable across various cell types, including filamentous fungi, yeasts, bacteria, and animal cells (Blagović et al. 2005; Ejsing et al. 2009; Stübiger et al. 2016; Walczak-Skierska et al. 2023). Two other methods for lipid extraction include the Matyash method (Matyash et al. 2008) and the Alshehry method (Alshehry et al. 2015). However, there is no significant difference in efficacy among these methods, as they can extract various lipid classes (Wong et al. 2019). We chose the Folch method for this study because the extracted lipids are located in the lower chloroform-organic phase, separated from the upper aqueous phase containing sugars, peptides, and amino acids by a distinct white-brown layer of hyphal debris. This feature facilitated more efficient and rapid isolation of extracted lipids compared to the Matyash method, which lacks a hyphal debris layer as a border marker, and the Alshehry method, which produces a lipid-containing monophase requiring further purification.

Notably, lipids have small molecular weights ranging from 400 to 1200 Da, so the matrix signal must be as small as possible to avoid overlapping with lipid peaks. The matrix 2,5-DHB was selected because it produced the smallest crystals and thinnest layers (Schiller et al. 2007). Moreover, 2,5-DHB emerged as the superior matrix for the MALDI-TOF MS technique due to its highly effective ion generation (Hsu et al. 2014). Additionally, to enhance accuracy, identifying unknowns requires testing a sample in at least 4 replications (Normand et al. 2017). In this study, we adopted the Folch method combined with the 2,5-DHB matrix to extract and analyze *P. insidiosum* lipid samples in 5 replicates using the MALDI-TOF MS assay to ensure highly accurate results.

### In-house *P. insidiosum* lipid profile database and the cutoff score

The MALDI-TOF MS analyses produced MSPs from 30 *P. insidiosum* isolates, classified in clade-I (n = 8), clade-II (n = 11), and clade-III (n = 11), as well as control organisms comprising various fungi (n = 50) (Tables 1 and 2). Since the clade IV strains of *P. insidiosum* are mostly clinically irrelevant and unavailable, they were excluded from this study. The obtained MSPs were used to create an in-house *P. insidiosum* lipid profile database and establish a cutoff value for pathogen identification. An identification score, typically ranging from 0 to 3 (Schulthess et al. 2014), is calculated based on the MSP of a test organism mapped against that of a database organism. As such, individual MSPs of all 50 control fungi (as test organisms) were mapped against those of all 30 *P. insidiosum* isolates (as database organisms), resulting in identification scores ranging from 1.745 to 2.512 (Table 2). The highest identification score (2.512) was obtained by mapping *Sarocladium* species strain RA076 with *P. insidiosum* strain Pi083C3. Self-mappings of MSPs among *P. insidiosum* (test and database strains are the same) showed identification scores ranging from 2.740 to 2.844, which were expectedly high. When considering only non-self-mappings where test and database strains of *P. insidiosum* differ, the identification scores ranged from 2.682 to 2.803 (Table 1).

Establishing a cutoff identification score is crucial for distinguishing whether a particular organism is *P. insidiosum*. Among various fungal species (Table 2), no fungus had an identification score higher than 2.512, indicating that this value could serve as the cutoff point for fungi. Therefore, a test sample with a score of 2.512 or less is likely to be a fungal species. On the other hand, within the *P. insidiosum* database group, all non-self-mappings had an identification score of at least 2.682, significantly higher than the cutoff point for fungi (2.512). Thus, a score of 2.682 is considered the lowest margin for confidently identifying an unknown organism as *P. insidiosum*, based on the extensive strain diversity of this pathogen included in the study (Table 1). However, an organism with an identification score falling between these discrimination points (2.512 and 2.682) was thus disqualified for either fungi or *P. insidiosum* and could be represented as other oomycetes. This discovery could potentially contribute to developing an effective diagnostic tool and strategy for *P. insidiosum*, thus advancing clinical diagnostics.

The cutoff identification score for lipids (2.682) in this study was found to be higher than that for proteins (2.00) for *P. insidiosum*-specific identification using MALDI-TOF MS analysis (Krajaejun et al. 2018). This stricter lipid score compared to proteins may be due to the fact that cellular lipids, which primarily form membranes and are structurally consistent, are less diverse than cellular proteins, which have a wide range of functions, including ribosomal proteins, housekeeping proteins, enzymes, and regulatory proteins (Singhal et al. 2015; Solntceva et al. 2020).

### Validation of the MALDI-TOF MS cutoff score for identifying *P. insidiosum*

MSPs generated from 25 blinded and previously untested lipid samples, including 10 *P. insidiosum* isolates and 15 other microorganisms (10 fungi and 5 oomycete species) (Table 3), were used to assess the detection performance of the MALDI-TOF MS assay. With the defined cutoff score (2.682), the assay correctly identified all *P. insidiosum* samples, achieving 100% detection sensitivity, making the assay an excellent choice for screening the pathogen. However, for detection specificity, the assay showed scores (ranging from 1.687 to 2.488) below the cutoff for all fungi and 2 oomycetes, namely *Pythium rhizo-oryzae* (score: 2.663) and *Paralagenidium karlingii* (score: 2.610). The organisms incorrectly identified as *P. insidiosum* included *Pythium catenulatum* (n = 2; scores: 2.718 and 2.720) and *Pythium aphanidermatum* (n = 1; score: 2.689), resulting in a detection specificity of 80%. The assay accuracy, representing the proportion of correct results (i.e., true positives and negatives), was 88%. It is important to note that all blinded samples prepared from fungi provided an identification score of 2.488 or less, which is compatible with a fungal identity (≤ 2.512) and significantly below the *P. insidiosum* cutoff value (2.682) or even the scores from other oomycetes (2.610–2.720; Table 3). In summary, the lipid profiles obtained from MALDI-TOF MS were able to differentiate *P. insidiosum* from the fungi.

We investigated further to understand the meaningfulness of the limited detection specificity of the lipid-based MALDI-TOF MS assay. All misidentified organisms (i.e., *P. catenulatum* and *P. aphanidermatum*) are oomycetes and closely related to *P. insidiosum* (Table 3). *P. catenulatum* is a common oomycete in a watery environment (Mar Htun et al. 2021). It has never been reported as a pathogen to humans or animals and is considered clinically irrelevant and unlikely to be isolated in a clinical microbiology laboratory. On the other hand, *P. aphanidermatum* is another oomycete species that could cause severe infection in humans, but to a much lower prevalence, with only 3 reported cases compared to at least 4,203 cases for *P. insidiosum* (Thongsuk et al. 2021; Yolanda and Krajaejun 2022). Therefore, an identification score of 2.682 or above can indicate the presence of a *Pythium* species, almost exclusively *P. insidiosum*, suggesting that the limited detection specificity should not be a significant clinical concern. For samples with an identification score between 2.512 and 2.682, a non-fungus and non-*P. insidiosum* organism can be expected. Two other oomycetes, *P. rhizo-oryzae* and *P. karlingii*, fell into this category (Table 3). Like *P. catenulatum*, *P. rhizo-oryzae* is typically an environmental non-pathogenic oomycete species that is clinically irrelevant and not isolated in clinical laboratories (Mar Htun et al. 2021). However, *P. karlingii* has been reported as an animal pathogen (White et al. 2020). Therefore, the lipid-based MALDI-TOF MS assay could provide clues to identify an unusual pathogenic oomycete causing a disease in humans or animals.

### Reproducibility and stability of the extracted lipids

Fungal identification based on morphology can generally be done when the organism is 7 days old to properly assess its microbiological characteristics (Morris et al. 1996). For the proteomic approach using MALDI-TOF MS analysis, a fungus of interest should be cultured for a minimum of 5 days. It is important to consider the duration of culture and growth conditions as they influence protein expression and the protein mass spectra, which can lead to unreliable interpretation of results (Lau et al. 2013). However, cellular lipids are relatively stable with distinct differences, making them a potential biomarker (van Meer et al. 2008). This study compared the lipids extracted from *P. insidiosum* at different ages, times, culture conditions, and storage durations to investigate whether these factors could affect the lipid mass spectra and, consequently, the identification of the organism.

Lipid samples were extracted from 3 *P. insidiosum* strains for reproducibility assessment. The organisms were grown at the same incubation temperature (25 °C) but with different culture media (i.e., SDA and BHI) and durations (i.e., 3, 5, and 7 days). In the reproducibility analysis, when the database strains (Pi009C1, Pi085C2, and Pi083C3) were grown on 1% SDA at 25 °C for 7 days for the second time, the obtained lipid extracts provided averaged identification scores ranging from 2.714 to 2.756, which were comparable to the first-time extraction (range: 2.718–2.763; Table 4). The experiment was repeated using a shorter colony incubation period (i.e., 5 and 3 days) before proceeding to the lipid extraction. These growth conditions resulted in identification scores of 2.719–2.747 for 5-day incubation and 2.745–2.764 for 3-day incubation, equivalent to the 7-day incubation (Table 4). All resulting identification scores were above the cutoff value (2.682), thus correctly determining all tested subject organisms as *P. insidiosum*. This evidence suggests that the incubation period for growing the organism on SDA can be shortened to 3 days to retain a compatible lipid-based identification score for specifically identifying *P. insidiosum*. However, when substituting the culture media from SDA to BHI for the same set of organisms, the obtained identification scores were below the cutoff, which was incompatible with *P. insidiosum* **(**Table 4). Therefore, the type of culture media is critical for generating a reproducible lipid extract to ensure an accurate result readout. To further ensure consistency and reproducibility in the lipid extraction step, an internal control sample using a known organism (such as a typed strain of *P. insidiosum*, another oomycete, or a filamentous fungus) can be included in the lipid-based MALDI-TOF assay.

**Table 4:** Identification scores of lipid samples extracted from 3 *Pythium insidiosum* strains grown at 25 °C on different culture conditions

Strains	Identification scores at different culture conditions (± SD)^a^				
	1% SDA, 7 days (1st extraction)	1% SDA, 7 days (2nd extraction)	1% SDA, 5 days	1% SDA, 3 days	BHI agar, 7 days
Pi009C1	2.718 (± 0.008)	2.731 (± 0.033)	2.719 (± 0.007)	2.745 (± 0.013)	2.594 (± 0.107)
Pi085C2	2.724 (± 0.036)	2.714 (± 0.035)	2.733 (± 0.011)	2.748 (± 0.035)	2.676 (± 0.023)
Pi083C3	2.763 (± 0.013)	2.756 (± 0.011)	2.747 (± 0.011)	2.764 (± 0.013)	2.660 (± 0.018)

^a^Each identification score is calculated as the average value from 5 replicates of the lipid samples, along with the standard deviation

Lipids were extracted from two strains of *P. insidiosum* and one strain of *P. rhizo-oryzae*, which were grown on 1% SDA at 25 °C for 7 days (Table 5). The lipid extracts were immediately aliquoted and stored at − 30 °C for 0, 14, 21, and 42 days, and then subjected to MALDI-TOF MS analysis against the in-house *P. insidiosum* lipid profile database to obtain identification scores for assessing sample stability. It was observed that the identification scores gradually decreased as the lipid sample was stored for a longer duration (Table 5). For example, the sample from *P. insidiosum* strain UN-10 provided an identification score of 2.766 (0 days of storage), 2.706 (14 days), 2.659 (21 days), and 2.498 (42 days). This inverted correlation was also noted in the other samples extracted from *P. insidiosum* strain UN-11 and *P. rhizo-oryzae strain* UN-08. Based on the cutoff threshold (2.682), the correct identifications to determine whether the organism is *P. insidiosum* were observed in the lipid samples stored at − 30 °C for up to 14 days; otherwise, identification of *P. insidiosum* to other species could occur. This observation aligns with Ulmer et al. (2021), who recommend storing lipid samples at − 20 °C for a short duration or frozen in liquid nitrogen for longer. Additionally, lipid extracts should be collected in an organic solvent (i.e., chloroform) and stored in an airtight container without exposure to light or oxygen to minimize degradation. For *P. insidiosum*, low-temperature storage (i.e., − 30 °C) could preserve a lipid sample for a week or two, and a longer storage duration could affect sample stability, leading to a false negative report.

**Table 5:** Identification scores of 3 *Pythium insidiosum*-extracted lipid samples stored at − 30 °C for different durations

Microorganisms	Identification scores at different storage durations (± SD)^a^			
	0 day	14 days	21 days	42 days
*P. insidiosum* UN-10	2.766 (± 0.010)	2.706 (± 0.003)	2.659 (± 0.018)	2.498 (± 0.130)
*P. insidiosum* UN-11	2.750 (± 0.015)	2.745 (± 0.027)	2.701 (± 0.009)	2.608 (± 0.007)
*P. rhizo-oryzae* UN-08	2.641 (± 0.020)	2.422 (± 0.113)	2.416 (± 0.073)	2.410 (± 0.041)

^a^Each identification score is calculated as the average value from 5 replicates of the lipid samples, along with the standard deviation

### Lipid-based biotyping of *P. insidiosum*

**Fig. 1:**
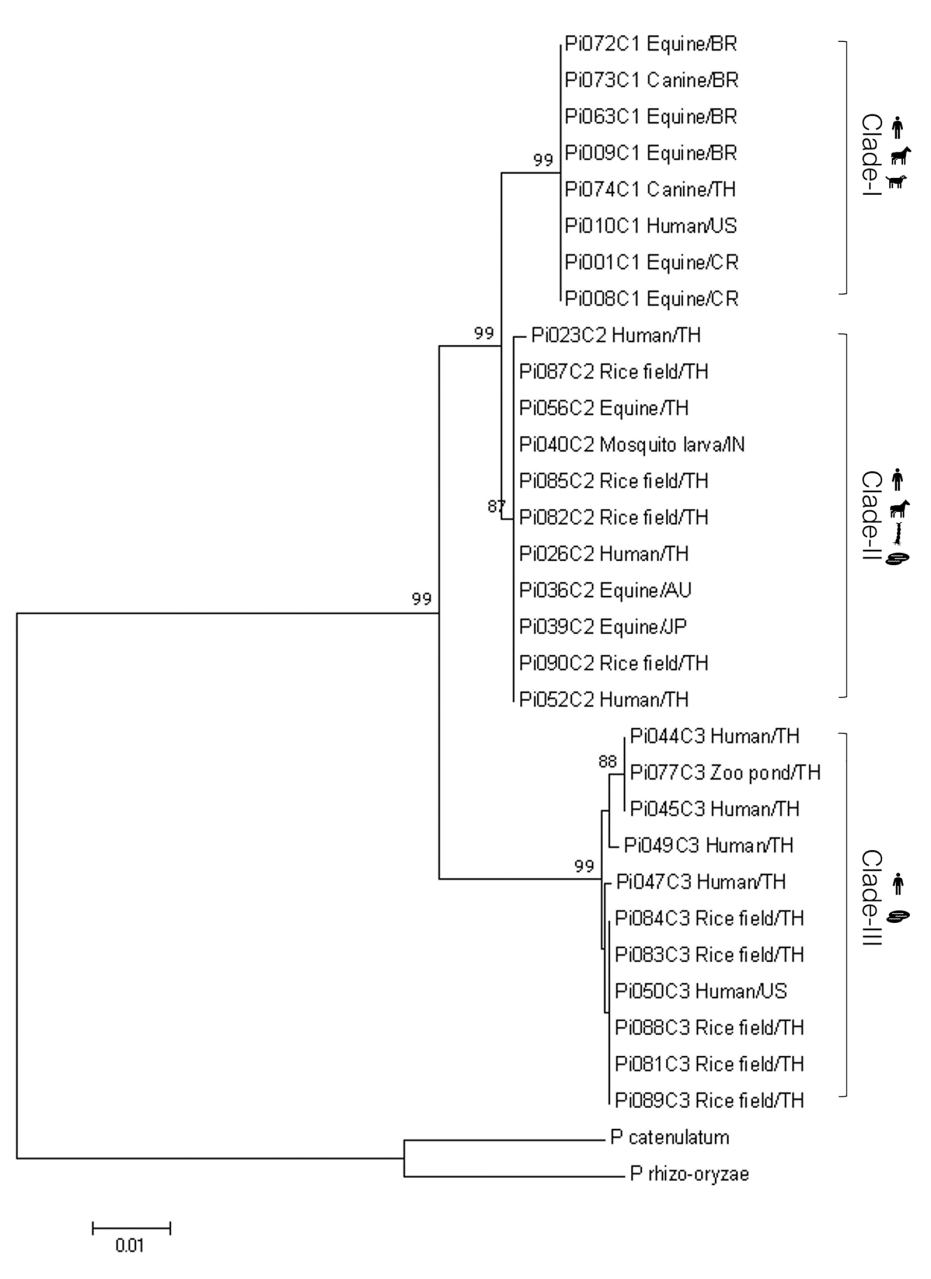
Phylogenetic analysis of *P. insidiosum* based on the rDNA ITS gene. The phylogenetic tree created using a neighbor-joining algorithm shows 3 genotypes of *P. insidiosum*: Clade-I, -II, and -III. All recruited strains are displayed with their sources and countries. *P. catenulatum* and *P. rhizo-oryzae* are presented as outgroups

Thirty isolates of *P. insidiosum* (Table 1) and 2 other *Pythium* species (*P. catenulatum* and *P. rhizo-oryzae*; served as the outgroup) isolates were classified based on their rDNA ITS sequences, and a phylogenetic tree was created using the neighbor-joining algorithm. *Pythium insidiosum* can be divided into 3 genotypes: Clade-I, -II, and -III (Fig. 1). The Clade-I strains were closely related to the Clade-II strains, sharing 91.2–97.2% nucleotide identity. The Clade-III strains shared 84.8–93% and 86.4–93.7% nucleotide identity with the Clade-I and Clade-II strains, respectively. Each genotype showed a geographical distribution pattern: Clade-I strains were found in the Americas and Thailand, Clade-II strains in Australia and Asia (including Thailand), and Clade-III strains in North America and Thailand (Table 1).

**Fig. 2:**
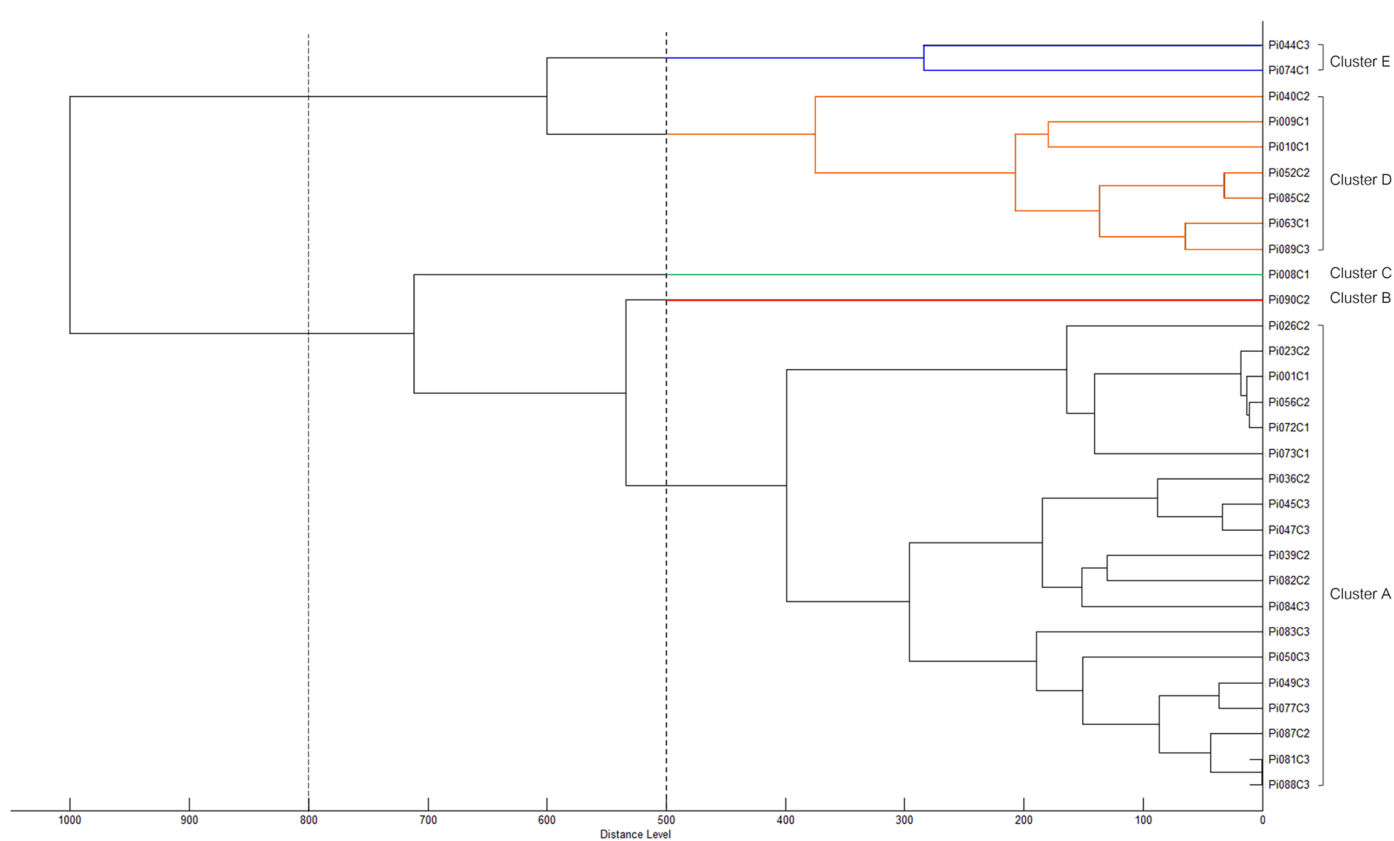
Lipid main spectral profile (MSP)-based hierarchical cluster analysis of *P. insidiosum*. The organisms are divided into 5 clusters (Clusters A, B, C, D, and E) when the distance level is set at 500 and 2 clusters (Clusters ABC and DE) when the distance level is set at 800

The lipid-derived MSPs from the same *P. insidiosum* isolates were analyzed using hierarchical cluster analysis. At a distance level of 500, the organisms were divided into 5 clusters: Clusters A (n = 19), B (n = 1), C (n = 1), D (n = 7), and E (n = 2), as shown in Fig. 2. When the distance level was increased to 800, the organisms were grouped into 2 clusters: Clusters ABC (n = 21) and DE (n = 9). The majority of the organisms fell into Cluster A or ABC. Regardless of the distance level cutoff, the MSP-based clusters included a mix of *P. insidiosum* isolates from rDNA ITS-based genotypes Clade-I, -II, and -III. This suggests that the lipid components can differentiate *P. insidiosum* into a distinct classification system that differs from gene-based biotyping (genotyping), where the classification is associated with the geographical origins of the organisms.

### Principal component analysis (PCA) of *P. insidiosum* lipid profiles

**Fig. 3:**
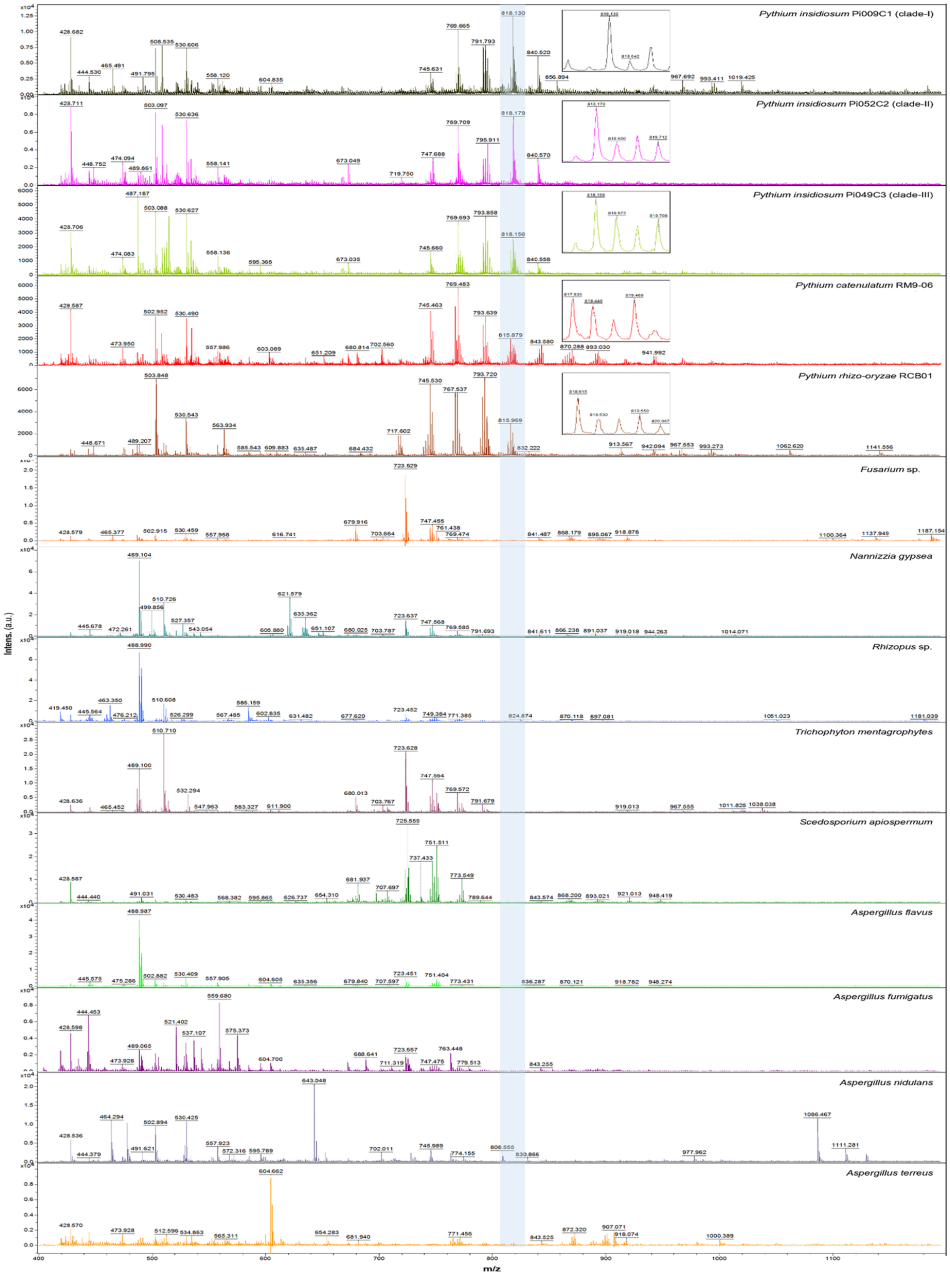
Lipid mass spectra of *P.* insidiosum, other Pythium species, and various fungi subjected to principal component analysis (PCA). *P. insidiosum* strains Pi009C1, Pi052C2, and Pi049C3 represent genotype Clade-I, -II, and -III, respectively. The 818 m/z peak (gray box) is specific to Pythium species. The Y-axis indicates mass intensities (in arbitrary units), while the X-axis indicates the mass range of lipids (400–1,200 m/z Da)

**Fig. 4:**
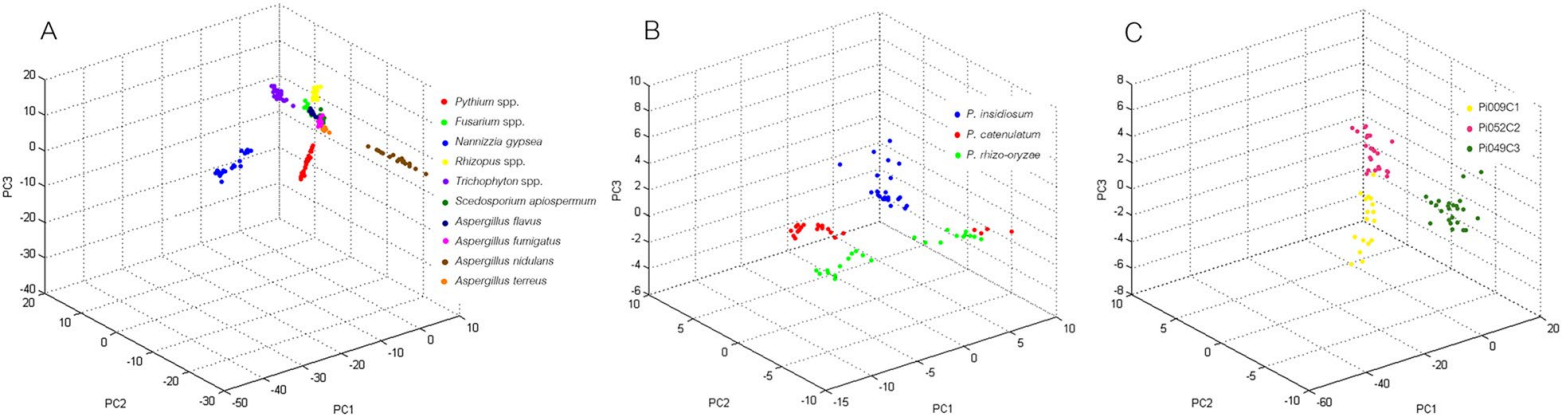
Principal component analysis (PCA) of lipid profiles from *P. insidiosum,* other *Pythium* species, and various fungi. **A** Differentiation of *Pythium species* (including *P. insidiosum*) and fungi from 6 genera (i.e., *Fusarium*, *Rhizopus*, *Trichophyton*, *Nannizzia*, *Scedosporium*, and *Aspergillus*); **B** Differentiation of *P. insidiosum*, *P. catenulatum*, and *P. rhizo-oryzae*; **C** Differentiation of *P. insidiosum* genotypes Clade-I (strain Pi009C1), -II (strain Pi052C2), and -III (strain Pi049C3)

Differences in lipid mass spectra (Fig. 3) were explored using PCA. The accuracy of the results was estimated using recognition capacity (RC) and cross-validation (CV) values from three statistical models: genetic algorithm (GA), supervised neural network (SNN), and quick classifier (QC). The GA model provided the highest RC and CV values and demonstrated that lipid components could differentiate the organisms at the genera, species, and genotype levels (Fig. 4). Based on PCA at the genus scale, *Pythium* species (*P. insidiosum, P. catenulatum*, and *P. rhizo-oryzae*) were grouped together, separated from other fungi such as *Fusarium* spp., *Rhizopus* spp., *Trichophyton* spp., *Nannizzia gypsea*, *Scedosporium apiospermum*, *Aspergillus flavus*, *Aspergillus fumigatus*, *Aspergillus nidulans*, and *Aspergillus terreus* (Fig. 4A). This organism differentiation was based on 10 lipid mass peaks and high PCA accuracy (99.70% RC and 97.62% CV) (Table 6). Notably, the 818.89 m/z peak appeared specific to *Pythium* species (Fig. 3).

**Table 6:** Lipid mass peaks for discriminating *Pythium* species from other fungi by using the genetic algorithm (GA) analysis

Mass peak (m/z; Da)	Start mass (m/z; Da)	End mass (m/z; Da)	Organisms									
			*Pythium* spp.^a^ (n = 33)	*Fusarium* spp. (n = 10)	*Nannizzia* *gypsea* (n = 1)	*Rhizopus* spp. (n = 3)	*Trichophyton* spp. (n = 3)	*Scedosporium* *apiospermum* (n = 5)	*Aspergillus* *flavus* (n = 5)	*Aspergillus* *fumigatus* (n = 5)	*Aspergillus* *nidulans* (n = 1)	*Aspergillus* *terreus* (n = 2)
488.00	487.55	488.38	** + **^b^	**−**	** + **	** + **	** + **	** + **	**−**	** + **	**−**	**−**
504.79	504.30	505.23	** + **	**−**	**−**	** + **	**−**	**−**	**−**	**−**	** + **	**−**
605.65	605.22	606.24	**−**	**−**	** + **	** + **	**−**	** + **	** + **	** + **	**−**	** + **
721.50	720.89	722.01	**−**	** + **	**−**	**−**	** + **	** + **	** + **	** + **	**−**	**−**
723.52	722.92	724.05	**−**	** + **	** + **	** + **	** + **	** + **	** + **	** + **	**−**	**−**
737.44	736.56	737.95	**−**	** + **	**−**	** + **	** + **	** + **	**−**	** + **	**−**	**−**
747.44	746.89	747.96	** + **	** + **	** + **	** + **	** + **	** + **	** + **	** + **	**−**	**−**
767.43	766.83	767.95	** + **	** + **	**−**	** + **	** + **	** + **	**−**	**−**	** + **	** + **
789.55	788.55	790.62	** + **	**−**	**−**	** + **	**−**	**−**	**−**	**−**	**−**	**−**
818.89	818.45	819.52	** + **	**−**	**−**	**−**	**−**	**−**	**−**	**−**	**−**	**−**

^a^*Pythium* spp. consist of *P. insidiosum*, *P. catenulatum*, and *P. rhizo-oryzae*

^b^“ + ” indicates the presence of the peak, while “**−**” indicates the absence of the peak

In the PCA analysis of *Pythium* species, it was observed that the lipid profiles of *P. insidiosum* (n = 30) were notably different from those of *P. catenulatum* (n = 2) and *P. rhizo-oryzae* (n = 1) (Fig. 4B). This differentiation was confirmed by high RC (100%) and CV (97.22%) values. Seven specific lipid mass peaks (419.81, 483.25, 566.80, 742.45, 769.49, 815.90, and 817.95 m/z) were crucial in distinguishing these species. The absence of 3 specific peaks (483.25, 566.80, and 742.45 m/z) indicated *P. insidiosum* (Table 7). Regarding genotypes, the PCA analysis provided enough resolution to categorize *P. insidiosum* into 3 clades **(**Fig. 4C). The PCA spots of genotype Clade-I and Clade-II were closer in distance than Clade-III, which aligns with the findings from the rDNA ITS-based phylogenetic tree (Fig. 1). Table 8 presents 10 lipid mass peaks crucial for this grouping outcome. The absence of the 563.84 m/z peak was associated with the clade-II genotype, while the absence of all the 807.72, 813.80, and 941.97 m/z peaks indicated the clade-III genotype. As a diagnostic application, the pattern of lipid peaks (Fig. 3) and PCA spot coordinates (Fig. 4) could provide additional evidence, alongside the identification score, to determine the identity of an organism at the genus, species, and genotype levels.

**Table 7:** Lipid mass peaks for discriminating *P. insidiosum*, *P. catenulatum*, and *P. rhizo-oryzae* by using the genetic algorithm (GA) analysis

Mass peak (m/z; Da)	Start mass (m/z; Da)	End mass (m/z; Da)	*Pythium* species		
			*P. insidiosum* (n = 30)	*P. catenulatum* (n = 2)	*P. rhizo-oryzae* (n = 1)
419.81	419.19	420.20	**−**^a^	+	**−**
483.25	482.31	483.63	**−**	+	+
566.80	566.36	567.37	**−**	+	+
742.45	741.96	742.52	**−**	+	+
769.49	768.93	770.02	+	+	+
815.90	815.35	816.51	+	+	+
817.95	817.38	818.51	+	+	+

^a^“ + ” indicates the presence of the peak, while “**−**” indicates the absence of the peak

**Table 8:** Lipid mass peaks for discriminating *P. insidiosum* genotypes Clade I, II, and III, by genetic algorithm (GA) analysis

Mass peak (m/z; Da)	Start mass (m/z; Da)	End mass (m/z; Da)	*P. insidiosum* genotypes		
			Clade I (n = 8)	Clade II (n = 11)	Clade III (n = 11)
563.84	563.49	564.21	+ ^a^	**−**	+
672.83	672.25	673.33	+	+	+
702.06	701.55	702.64	+	+	+
765.41	764.52	765.92	+	+	+
767.43	766.78	767.96	+	+	+
795.64	795.10	796.18	+	+	+
807.72	806.59	808.24	+	+	**−**
813.80	812.67	814.69	+	+	**−**
817.91	817.32	818.45	+	+	+
941.97	941.31	942.58	+	+	**−**

^a^“ + ” indicates the presence of the peak, while “-” indicates the absence of the peak

## Conclusions

*P. insidiosum* is distributed worldwide and causes a life-threatening disease (pythiosis) in humans and animals. Early and effective diagnosis is critical for disease management. The current study introduces an innovative approach to identifying *P. insidiosum* by utilizing MALDI-TOF MS lipid profiling, offering high detection performance. The Folch method and the 2,5-DHB matrix enabled the extraction and analysis of lipid samples with high efficiency, as demonstrated by generating unique MSPs from *P. insidiosum*. These profiles form a novel in-house lipidomic database, which aids in developing cutoff identification scores that differentiate *P. insidiosum* from other fungal organisms: scores ≥ 2.682 indicated *P. insidiosum*, scores ≤ 2.512 suggested fungi, and scores in between pointed to other oomycetes. This diagnostic approach showed high detection sensitivity (100%), specificity (80%), and accuracy (88%) in identifying *P. insidiosum*. The limited specificity in detection was due to false positive samples from closely related *Pythium* species, which are not a significant clinical concern. The influence of culture conditions, incubation periods, and storage on the accuracy of lipid mass spectra was relatively minimal, offering significant advantages in sample preparation, stability, and reproducibility over protein profile-based methods. Furthermore, exploring lipid MSPs through the hierarchical cluster analysis led to an alternative classification system for potentially investigating the epidemiological pattern and clinical correlation of *P. insidiosum*.

In summary, utilizing MALDI-TOF MS for the lipid profiling of *P. insidiosum* enhances diagnostic efficiency and opens up new microbial identification and classification possibilities. This advancement holds promise for improving clinical outcomes through timely and accurate diagnosis, emphasizing the critical role of advanced technology in microbial research and diagnostics. Future studies are encouraged to further broaden, adapt, and validate this lipid profile application as an emerging diagnostic technique for microbial identification.

## Abbreviations

DHB: Dihydroxybenzoic acid
MSP: Main spectral profile
PCA: Principal component analysis
GA: Genetic algorithms
SNN: Supervised neural network
QC: Quick classifier
RC: Recognition capacity
CV: Cross-validation value
